# Recurrence of Phospholipase A2 Receptor–Associated Membranous Nephropathy in the Absence of Serum Anti-Phospholipase A2 Receptor Antibodies Reappearance

**DOI:** 10.1016/j.ekir.2024.12.009

**Published:** 2024-12-10

**Authors:** Ilias Bensouna, Marion Delafosse, Claire Cartery, Thomas Stehlé, Clementine Rabate, Fanny Boullenger, David Buob, Jean-Jacques Boffa, Karine Dahan, Emmanuel Esteve

**Affiliations:** 1 Nephrology and Dialysis Department, Hôpital Tenon, Assistance Publique—Hôpitaux de Paris, Paris, France; 2 Nephrology Department, Centre hospitalier de Valenciennes, Valenciennes, France; 3 Service de Néphrologie et Transplantation, Hôpitaux Universitaires Henri Mondor, Fédération Hospitalo-Universitaire (Innovative Therapy for Immune Disorders), Assistance Publique des Hôpitaux de Paris, Créteil, France; 4 Institut Mondor de Recherche Biomédicale, Institut National de la Santé et de la Recherche Médicale, Université Paris Est Créteil, Créteil, France; 5 Division of Nephrology and Dialysis, Hôpital privé Claude Galien, Ramsay Santé, Quincy-Sous-Sénart, France; 6 Nephrology Department, Centre hospitalier intercommunal André Grégoire, Montreuil, France; 7 Pathology Department, Hôpital Tenon, Assistance Publique—Hôpitaux de Paris, Paris, France; 8 INSERM U1155 CORAKID, Sorbonne University, Paris, France

**Keywords:** anti-PLA2R antibody, idiopathic membranous nephropathy, relapse, seronegative

## Introduction

The past 15 years of research on the pathogenic role of antiphospholipase A2 receptor (PLA2R) antibodies has significantly transformed nephrologists’ approach to membranous nephropathy (MN). Offering nearly 100% specificity, a positive PLA2R serology is enough to confirm the diagnosis of MN. Beyond diagnosis, anti-PLA2R serology has emerged as a potent prognostic biomarker, leading to the integration of anti-PLA2R titers in treatment decision algorithms.^1^ In PLA2R-associated MN, immunological remission precedes clinical remission, making it a precocious surrogate end point. Persistent positive or reappearance of anti-PLA2R antibody is highly predictive of relapse. However, anti-PLA2R sensitivity is not as perfect as its specificity.^2^ In a Chinese retrospective cohort of 514 histologically proven PLA2R-associated MN, 127 patients (24.8%) were seronegative. Seronegative patients tend to have lower proteinuria, higher albuminemia, and estimated glomerular filtration rate, and are more likely to reach complete remission.^3^ Although well-characterized during the disease’s first manifestation, the literature lacks comprehensive descriptions of patients experiencing relapse in the absence of detectable circulating anti-PLA2R antibodies, with only a recent case report available.^4^^,^^5^

## Results

**Figure 1:**
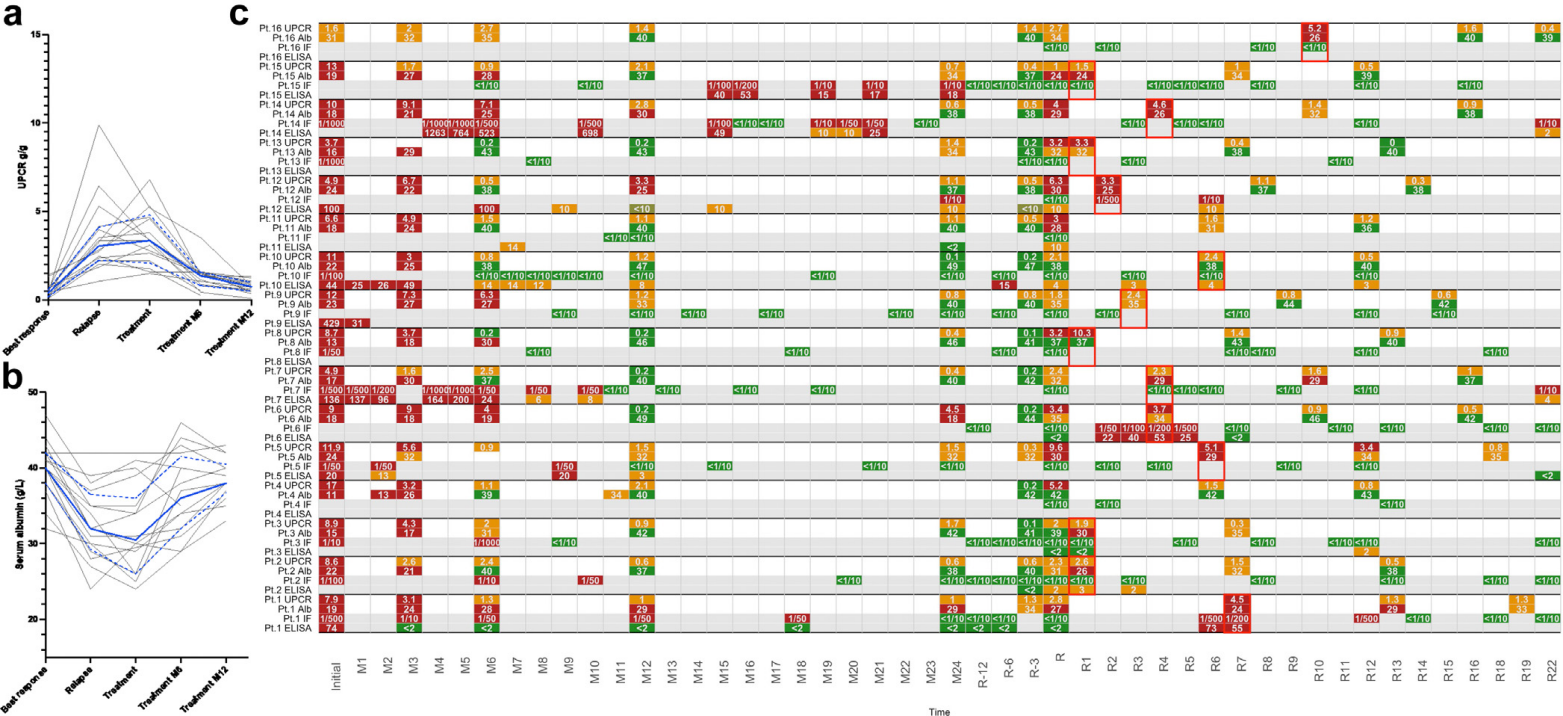
Biological and immunological presentation of patients with MN with seronegative relapse. Evolution of (a) UPCR and (b) serum albumin level at baseline, clinical relapse, and treatment, 6 months and 12 months after treatment for each patient. Independent patient trajectories are represented by grey lines. Median is represented by the single blue line, whereas interquartiles are represented by dotted blue lines. (c) Proteinuria (UPCR, g/g), serum albumin (g/l), and anti-PLA2R antibody levels measured by IIF assay and ELISA (gray lines) are shown at the initial flare and during follow-up, up to 24 months postflare (M24). Additional time points include 12, 6, and 3 months before clinical relapse (R-12, R-6, R-3), at the time of clinical relapse (R), and up to 22 months after relapse (R-22). UPCR < 0.3 g/g and albumin > 35 g/l are indicated in green; UPCR between 0.3 and 3 g/g and albumin between 30 and 35 g/l have been indicated in yellow; and UPCR > 3 g/g and albumin < 30 g/l have been indicated in red. Seronegativity by IIF is shown in green, whereas seropositivity is shown in red. Anti-PLA2R titers measured by ELISA below the laboratory threshold of 14 RU/ml but above 2 RU/ml are represented in yellow. For patients receiving immunosuppressive therapy at clinical relapse, the month of treatment initiation is highlighted with a red frame. ELISA, enzyme-linked immunosorbent assay; IIF, immunofluorescence; PLA2R, phospholipase A2 receptor; UPCR, urinary protein-to-creatinine ratio.

We performed a retrospective analysis, identifying 16 patients who presented clinical relapses of MN between October 2016 and June 2022, without detectable circulating PLA2R antibodies, as assessed by indirect immunofluorescence assay (IIF). Further details are provided in Supplementary Methods and Supplementary Figure S1. Characteristics of relapsing patients with seronegative anti-PLA2R antibodies are reported in Table 1. Following a median interval of 65 months since the previous flare, all patients showed a significant increase in urinary protein-to-creatinine ratio, increasing from 0.43 (0.17–0.52) g/g prerelapse to 3.70 (2.41–5.16) g/g at relapse (*P* < 0.001). A urinary protein-to-creatinine ratio > 3.5 g/g was observed in 10 out of 16 patients. Serum albumin levels decreased from 40.0 (38.0–42.0) g/l to 29.5 (26.0–35.0) g/l (*P*-value < 0.001), and 5 of 16 patients had authentic nephrotic syndromes. Glomerular filtration rate was not significantly altered: 79 (55–115) ml/min per 1.73 m^2^ versus 84 (53–111) ml/min per 1.73 m^2^ before relapse. Individual trajectories are represented in Figure 1. When available, kidney biopsies (*n* = 8) at clinical relapse consistently demonstrated typical MN IgG and PLA2R-positive deposits (Supplementary Figure S2). Electron microscopy analysis could only be performed for one of these patients, revealing the concomitant presence of both ancient and recent deposits. In comparison with the initial biopsy, patients at relapse exhibited a higher frequency of stage 3 MN (6/8 [75%]; *P*-value = 0.018) and consistently demonstrated at least 1 globally sclerotic glomerulus (*P*-value = 0.007). A trend toward more frequent focal segmental glomerulosclerosis lesions (50% vs. 23%; *P*-value = 0.3) was observed (Supplementary Table S1). On top of IIF, enzyme-linked immunosorbent assay (ELISA) testing for anti-PLA2R antibodies was performed for 7 patients. All patients tested below the 14 RU/ml threshold (< 2 RU/ml in 3, 2 RU/ml in 1, 4 RU/ml in 1, and 10 RU/ml in 2 patients). In 3 patients, anti-PLA2R serology eventually became positive with IIF ratios between 1:50 and 1:500 and ELISA between 22 RU/ml and 75 RU/ml, 2 to 6 months after clinical relapse (Figure 1c).

**Table 1:** Patients’ characteristics at clinical relapse and 12 months post treatment introduction

Characteristic	Initial diagnosis	Relapse	M12	*P*-value^a^
Clinical status (*n* = 16)				
Gender^b^, *n* (%)				
Female	3 (18.8%)			
Male	13 (81.3%)			
Age (yr), *n* [IQR]	45.5 [29.5–62.5]			
Time to relapse (mo), *n* [IQR]	54 [41–73]			
Histology (*n* = 8)				
IgG+ extramembranous deposits, *n* (%)	8 (100.0%)			
PLA2R+ staining, *n* (%)	8 (100.0%)			
FSGS, *n* (%)	4 (50.0%)			
Treatment (*n* = 16)				
Treatment, *n* (%)				
Nephroprotective care	1 (6.3%)	2 (12.5%)		
Rituximab	11 (68.8%)	14 (87.5%)		
Corticosteroids + Cyclophosphamide	3 (18.8%)			
Corticosteroids + chloraminophene	1 (6.3%)			
Time to rituximab (mo) (*n* = 14), *n* [IQR]	4 [1–6]			
Biology (*n* = 16)				
eGFR (mmol/min per 1.73 m^2^), median [IQR]	74 [47–101]	87 [66–117]	82 [54–107]	0.9
albumin (g/l), median [IQR]	19.0 [16.8–22.3]	29.5 [26.0–35.0]	38.0 [35.8–40.0]	<0.001
UPCR (g/g), median [IQR]	8.26 [4.86–9.52]	3.70 [2.41–5.16]	0.69 [0.52–0.95]	<0.001
Remission (*n* = 16)				
Partial remission, *n* (%)			13 (81.3%)	
Complete remission, *n* (%)			3 (18.8%)	
Partial or complete remission, *n* (%)			16 (100.0%)	

eGFR, estimated glomerular filtration rate; FSGS, focal segmental glomerulosclerosis; IQR, interquartile range; PLAR2, antiphospholipase A2 receptor; UPCR, urinary protein-to-creatinine ratio.

a Wilcoxon rank sum test.

b Ascertained by self-report.

All the patients received supportive treatment and 14 were administered rituximab. Twelve months after treatment initiation, median urinary protein-to-creatinine ratio decreased from 3.70 (2.41–5.16) g/g to 0.69 (0.52–0.95) g/g (*P* value < 0.001) and median serum albumin increased from 29.5 (26.0–35.0) g/l to 38.0 (35.8–40.0) g/l (*P* value < 0.001) (Figure 1a and b). Estimated glomerular filtration rate remained stable. All patients reached partial remission, and 3 patients (all treated with rituximab) achieved complete remission. No severe adverse event (defined by any event that would lead to hospitalization, acute kidney failure or death) was reported during the median of 34 (15–58) months of follow-up. Seven out of 16 patients eventually reached complete remission (6 in the rituximab group, and 1 in the supportive care group). Five patients suffered another relapse after treatment within a median delay of 39 (30–44) months.

## Discussion

We report on a peculiar group of patients who, after successful treatment of PLA2R-associated MN, presented clinical relapse without detection of circulating anti-PLA2R antibodies by IIF at clinical relapse.

When considered individually, the proteinuria results could suggest focal segmental glomerulosclerosis scarring. However, the concomitant hypoalbuminemia; and the integration of these findings within the individual patients’ timelines, along with the pronounced impact of rituximab on the depicted curves in Figure 1, strongly support the presence of active immunological glomerular injuries. This is corroborated by the systematic positivity of the PLA2R glomerular staining whenever a biopsy was performed.

Failure to detect circulating anti-PLA2R could be explained by ELISA and IIF sensitivity limitations. Although Western blot is considered more sensitive, it is not feasible for routine clinical use and was unavailable in this retrospective study.^6^ IIF may be more sensitive than ELISA but is prone to interpretative bias, especially at low titers. One study showed that lowering the ELISA positivity threshold to 2.7 RU/ml increased sensitivity to 88% without affecting specificity (96%).^7^ However, Bobart *et al.* found that, among IIF-negative patients with ELISA results between 2 RU/ml and 20 RU/ml, MN was confirmed in only 48 of 80 (57.5%) biopsies.^8^ Given the high prevalence of relapse in patients with MN and the low likelihood of developing concurrent nephrotic disease, this reduction in specificity is unlikely to compromise the positive predictive value, thereby supporting consideration of a lower ELISA threshold for relapse diagnosis. Unfortunately, ELISA is not routinely performed in IIF-negative cases here, limiting our ability to determine if more than 4 of the 7 would have tested positive at a lower threshold. More frequent testing may allow for earlier detection, potentially explaining the 3 positive serologies observed during follow-up, although most of the cohort, including 4 on prolonged supportive care and 2 untreated, remained IIF-negative. The “glomerulus as a sink” hypothesis, which proposes that glomerular antigenic sites must be saturated before circulating antibodies can be detected, has been proposed to explain the discrepancies between serological and histological testing at the first flare.^9^ There is no reason why this hypothesis should not also apply to relapsing patients.

The active research around PLA2R-associated MN has demonstrated the direct nephrotoxicity they mediate. Relapsing patients have a higher risk of developing focal segmental glomerulosclerosis scars, remaining proteinuria, and progression toward CKD. In our opinion, the lack of detectable antibodies should not lead to delaying or avoiding immunomodulatory treatment, particularly during relapses. Large cohort-based risk stratification scores have allowed international consensus on initial treatment strategies.^1^ Efforts should now be focused on relapsing patients to enhance the prediction of spontaneous remission probability and long-term risks, enabling personalized evidence-based treatment strategies.

## Conclusion

Just as there are seronegative but glomerular positive PLA2R MN cases at diagnosis, we report on cases of apparently seronegative relapses by IIF and following the manufacturer’s ELISA threshold. Although PLA2R serology is highly performant, nephrologists should keep in mind that it does not have perfect sensitivity at diagnosis or in the context of a relapse and that albumin and proteinuria remain the most clinically relevant activity markers of MN. ELISA results under the 14 RU/ml threshold might indicate immunologically active disease and dual testing by IIF and ELISA could be interesting in challenging cases.

## Disclosure

All the authors declared no competing interests.

## Patient Consent

All patients received oral and written information and agreed to the use of their personal data as per the Helsinki principal and French regulation on retrospective non interventional medical research.

## Supplementary Material

Supplementary File (PDF) **Supplementary Methods. Figure S1.** Evolution of (A) urinary protein-to-creatinine ratio in g/g and (B) serum albumin (g/l). **Figure S2.** PLAR2 revealed on immunochemistry. **Table S1.** Histological characteristics at diagnosis and relapse. **STROBE Checklist.**
