# Supplementary material for: Recurrence of Phospholipase A2 Receptor–Associated Membranous Nephropathy in the Absence of Serum Anti-Phospholipase A2 Receptor Antibodies Reappearance
Source: Kidney Int Rep. 2024 Dec 10;10(3):952–5. doi: 10.1016/j.ekir.2024.12.009 (PMC11993193; doi:10.1016/j.ekir.2024.12.009)
Supplement: Supplementary File (PDF) — Supplementary Methods. Figure S1. Evolution of (A) urinary protein-to-creatinine ratio in g/g and (B) serum albumin (g/l). Figure S2. PLAR2 revealed on immunochemistry. Table S1. Histological characteristics at diagnosis and relapse. STROBE Checklist. [file mmc1.pdf]

Supplementary methods

We assessed all patients known to have relapsing PLA2R-associated membranous nephropathy by screening the registries and PLA2R results in identified clinical charts emerging from two centers of the "Centre de Référence Maladies Rare - Syndrome Néphrotique Idiopathique": Tenon Hospital in Paris, France, and Henri Mondor Hospital in Créteil, France. These registries are part of a French national initiative that collects data on patients with "idiopathic" nephrotic syndrome, including membranous nephropathy. All patients were followed (12/16) or referred (4/16) to one of these expert centers for tertiary expertise. To ensure exhaustivity in data collection attending nephrologist of the patients referred to the CRMR were directly contacted, and clinical charts retrieved from their respective health structure (André Grégoire (Montreuil, France), Valenciennes (Valenciennes, France), and Claude Gallien (Quincy-sous-Sénart, France)).

Clinical (demographic data, initial clinical presentation, treatment, remission and adverse effect), biological (eGFR, serum albumin, UPCR, anti-PLA2R titer) and histological (MN stage, IgG+ extramembranous deposits, PLA2R+ staining, FSGS lesions, glomerulosclerosis and interstitial fibrosis lesions) data were retrieved from clinical charts by IB. Initial diagnosis of primary PLA2R MN was based on the presence of circulating anti-PLA2R Ab (N = 14) and/or with histopathological exam (N = 15). The two patients without circulating anti-PLA2R Ab detection were diagnosed before circulating anti-PLA2R testing was available, so their diagnoses relied solely on histological examination. No patient had a negative serologic test or discordance between histology and serology. Clinical partial and complete remission are defined by urinary protein level between 0.3 and 3.5 g/g with a decrease by at least 50% from the initial value and < 0.3 g/g respectively, with stabilization or improvement in estimated glomerular filtration rate (eGFR). All patients included suffered clinical relapse without immunological relapse. Research of serum PLA2R Ab remained negative at the time of clinical relapse by indirect immunofluorescence assay (IIF), and eventually by enzyme-linked immunosorbent assay (ELISA) using a 14 relative units (RU)/mL positivity threshold, both tests developed by EUROIMMUN AG, Lübeck, Germany.

All statistics were performed using GraphPad Prism version 10.1.0 for MacOs (GraphPad Software, La Jolla, CA) and R statistical software version 4.3.0. Continuous variables are expressed as median (interquartile range [IQR]) and are compared using Wilcoxon rank sum test or Kruskal-Wallis followed by Dun’s multiple comparison test when appropriate. Categorical variables are compared using Fisher’s exact test.

Characteristic	**Initial**, N = 13[1]	**Relapse**, N = 8[1]	p-value[2]	N[3]
**MN staging**			0.018	21/21
1 or 2	11 (85)	2 (25)		
3	2 (15)	6 (75)		
**Presence of FSGS lesion**	3 (23)	4 (50)	0.3	21/21
% of affected glomeruli	7.0 (5.5 – 8.0)	20.0 (16.0 – 22.8)	0.057	7/21
**Global glomerulosclerosis**	5 (38)	8 (100)	0.007	21/21
% of affected glomeruli	13 (4 – 27)	15 (8 – 26)	0.7	13/21
**Presence of interstitial fibrosis**	7 (54)	4 (50)	>0.9	21/21
% of interstitial fibrosis	10.0 (10.0 – 13.8)	10.0 (10.0 – 10.0)	>0.9	11/21
**Presence of Arterial sclerosis**	6 (46)	4 (50)	>0.9	21/21

[1]n (%); Median (IQR)

[2]Fisher's exact test; Wilcoxon rank sum exact test; Wilcoxon rank sum test

[3]N not Missing/Total N

Supplementary Table S1: Histological characteristics at diagnosis and relapse

Among the 13 biopsies at initial flare, we found 2 stage 1 MN, 9 stage 2 MN and 2 stage 3 MN. Among the 8 biopsies at relapse, we found 1 stage 1 MN, 1 stage 2 MN and 6 stage 3 MN.

MN = membranous nephropathy; FSGS = focal segmental glomerulosclerosis

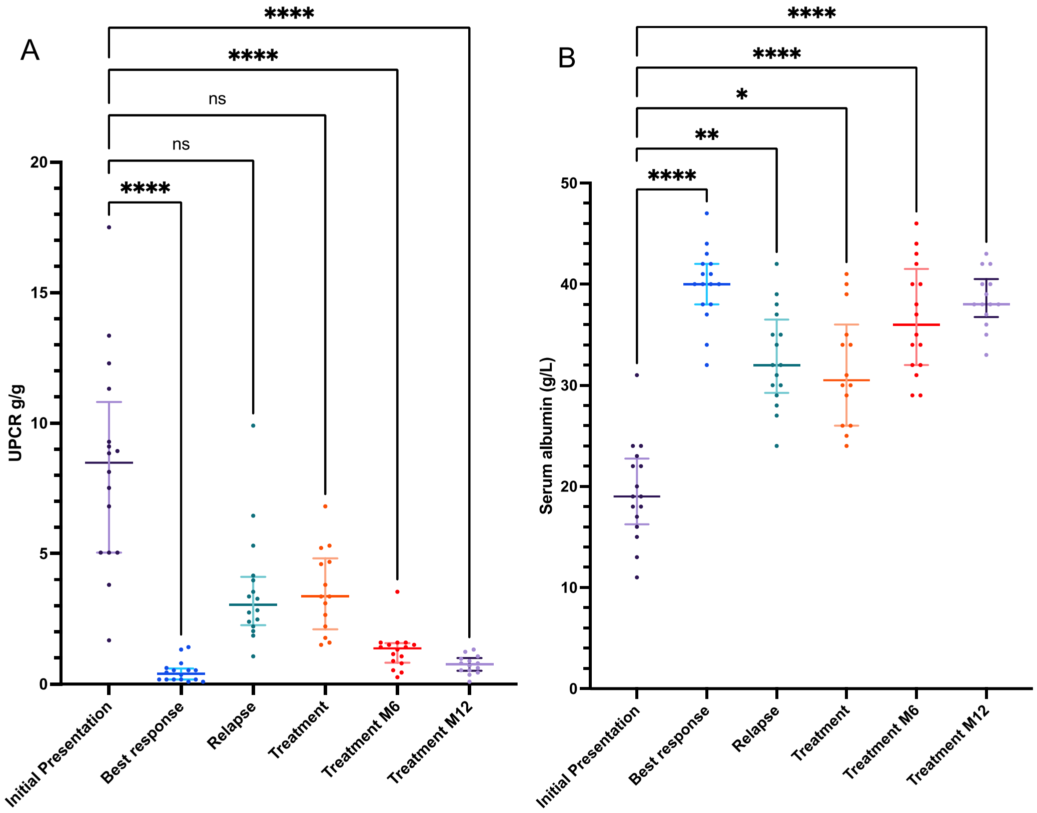

Supplementary Figure S1: Evolution of Urinary protein to creatinine ratio (A) in g/g and Serum Albumin (g/L) (B).

*Median and Interquartile Range, Kruskal-Wallis followed by Dunn's multiple comparison test. ns not significant *p value<0,05 **p value< 0,01 **** p-value < 0,0001*

A B

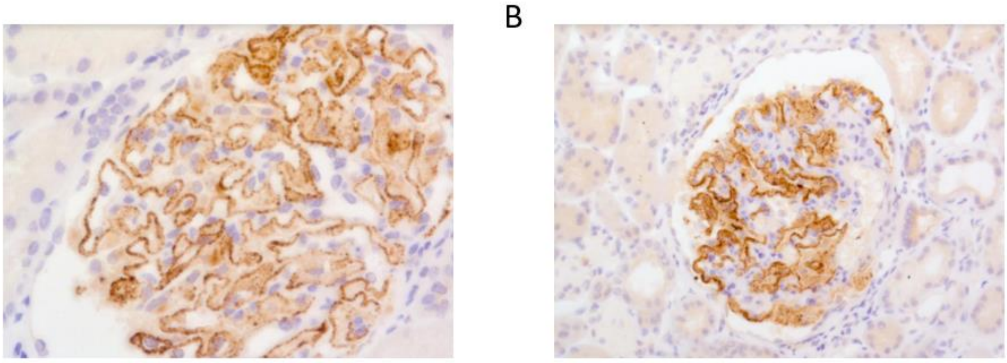

Supplementary Figure S2: PLAR2 revealed on immunochemistry

At initial diagnosis (A) and at clinical relapse (B)

STROBE Statement—checklist of items that should be included in reports of observational studies

	Item No.	Recommendation	Page No.	Relevant text from manuscript
**Title and abstract**	1	(*a*) Indicate the study's design with a commonly used term in the title or the abstract	N/A	
		(*b*) Provide in the abstract an informative and balanced summary of what was done and what was found	N/A	
**Introduction**				
Background/rationale	2	Explain the scientific background and rationale for the investigation being reported	2	The last 15 years of research on the pathogenic role of anti-phospholipase A2 receptor (PLA2R) antibodies has significantly transformed nephrologists' approach of membranous nephropathy (MN). Offering nearly 100% specificity, a positive PLA2R serology is enough to confirm the diagnosis of MN. Beyond diagnosis, anti-PLA2R serology has emerged as a potent prognostic biomarker, leading to the integration of anti-PLA2R titers in treatment decision algorithms.1 In PLA2R associated MN, immunological remission precedes clinical remission, making it a precocious surrogate endpoint. Persistent positive or reappearance of anti-PLA2R antibody is highly predictive of relapse. However, anti-PLA2R sensitivity is not as perfect as its specificity.2 In a Chinese retrospective cohort of 514 histologically proven PLA2R associated MN, 127 (24,8%) patients were seronegative. Seronegative

				patients tend to have lower proteinuria, higher albuminemia and eGFR and are more likely to reach complete remission.3 While well-characterized during the disease's first manifestation, the literature lacks comprehensive descriptions of patients experiencing relapse in the absence of detectable circulating anti PLA2R antibodies, with only a recent case report available.
Objectives	3	State specific objectives, including any prespecified hypotheses	2	We performed a retrospective analysis, identifying 16 patients who presented clinical relapses of MN [...] without detectable circulating PLA2R antibodies, as assessed by indirect immunofluorescence assay.
**Methods**				
Study design	4	Present key elements of study design early in the paper	Supp	Clinical (demographic data, initial clinical presentation, treatment, response and adverse effect), biological (eGFR, serum albumin, UPCR, anti-PLA2R titer) and histological (MN stage, IgG+ extramembranous deposits, PLA2R+ staining, FSGS lesions) data were retrieved from clinical charts by IB.
Setting	5	Describe the setting, locations, and relevant dates, including periods of recruitment, exposure, follow-up, and data collection	3 + supp	We performed a retrospective analysis, identifying 16 patients who presented clinical relapses of MN between October 2016 and June 2022, without detectable circulating PLA2R antibodies, as assessed by indirect immunofluorescence assay (IIF). All patients had prior diagnosis of PLA2R-associated MN [...]We assessed all patients known to have relapsing PLA2R-associated

				membranous nephropathy by screening the registries and PLA2R results in identified clinical charts emerging from two centers of the "Centre de Référence Maladies Rare - Syndrome Néphrotique Idiopathique": Tenon Hospital in Paris, France, and Henri Mondor Hospital in Créteil, France. These registries are part of a French national initiative that collects data on patients with "idiopathic" nephrotic syndrome, including membranous nephropathy. All patients were followed (12/16) or referred (4/16) to one of these expert centers for tertiary expertise. To ensure exhaustivity in data collection attending nephrologist of the patients referred to the CRMR were directly contacted, and clinical charts retrieved from their respective health structure (André Grégoire (Montreuil, France), Valenciennes (Valenciennes, France), and Claude Gallien (Quincy-sous-Sénart, France)).
Participants	6	(*a*) *Cohort study*—Give the eligibility criteria, and the sources and methods of selection of participants. Describe methods of follow-up<br>*Case-control study*—Give the eligibility criteria, and the sources and methods of case ascertainment and control selection. Give the rationale for the choice of cases and controls<br>*Cross-sectional study*—Give the eligibility criteria, and the sources and methods of selection of participants	Supp	. Initial diagnosis of primary PLA2R MN was based on the presence of circulating anti-PLA2R Ab (N = 14) and/or with histopathological exam (N = 15). [...]All patients included suffered clinical relapse without immunological relapse. Research of serum PLA2R Ab remained negative at the time of clinical relapse.

		(*b*) *Cohort study*—For matched studies, give matching criteria and number of exposed and unexposed<br><br>*Case-control study*—For matched studies, give matching criteria and the number of controls per case	NA	
Variables	7	Clearly define all outcomes, exposures, predictors, potential confounders, and effect modifiers. Give diagnostic criteria, if applicable	Supp	Clinical partial and complete remission are defined by urinary protein level between 0.3 and 3.5 g/g with a decrease by at least 50% from the initial value and < 0.3 g/g respectively, All patients included suffered clinical relapse without immunological relapse.
Data sources/ measurement	8*	For each variable of interest, give sources of data and details of methods of assessment (measurement). Describe comparability of assessment methods if there is more than one group	Supp	Clinical (demographic data, initial clinical presentation, treatment, response and adverse effect), biological (eGFR, serum albumin, UPCR, anti-PLA2R titer) and histological (MN stage, IgG+ extramembranous deposits, PLA2R+ staining, FSGS lesions) data were retrieved from clinical charts by IB [...]. Research of serum PLA2R Ab remained negative at the time of clinical relapse by both enzyme-linked immunosorbent assay (ELISA) using a 14 relative units (RU)/ml positivity threshold, and by indirect immunofluorescence assay (IIF), both tests developed by EUROIMMUN AG, Lübeck, Germany.
Bias	9	Describe any efforts to address potential sources of bias	3	Clinically relevant relapse was defined variably among referent nephrologists but consistently included at least a 50% rapid increase in urinary protein-creatinine ratio (UPCR).

Study size	10	Explain how the study size was arrived at	3	We performed a retrospective analysis, identifying 16 patients who presented clinical relapses of MN between October 2016 and June 2022

Continued on next page

Quantitative variables	11	Explain how quantitative variables were handled in the analyses. If applicable, describe which groupings were chosen and why	Supp	Continuous variables are expressed as median (interquartile range [IQR])
Statistical methods	12	(*a*) Describe all statistical methods, including those used to control for confounding	Supp	Continuous variables [...] are compared using Wilcoxon rank sum test or Kruskal-Wallis followed by Dun's multiple comparison test when appropriate. Categorical variables are compared using Fisher's exact test
		(*b*) Describe any methods used to examine subgroups and interactions	N/A	
		(*c*) Explain how missing data were addressed	N/A	
		(*d*) *Cohort study*—If applicable, explain how loss to follow-up was addressed<br>*Case-control study*—If applicable, explain how matching of cases and controls was addressed<br>*Cross-sectional study*—If applicable, describe analytical methods taking account of sampling strategy	N/A	
		(*e*) Describe any sensitivity analyses	N/A	
**Results**				
Participants	13*	(a) Report numbers of individuals at each stage of study—eg numbers potentially eligible, examined for eligibility, confirmed eligible, included in the study, completing follow-up, and analysed	3	We performed a retrospective analysis, identifying 16 patients who presented clinical relapses of MN between October 2016 and June 2022
		(b) Give reasons for non-participation at each stage	N/A	
		(c) Consider use of a flow diagram	N/A	
Descriptive data	14*	(a) Give characteristics of study participants (eg demographic, clinical, social) and information on exposures and potential confounders	3-4 + Table 1	Following a median interval of 65 months since the previous flare, all patients showed a significant rise in urinary protein-creatinine ratio

(UPCR), increasing from 0.43 g/g [0.17–0.52] pre-relapse to 3.70 g/g [2.41–5.16] at relapse ($p < 0.001$). A UPCR > 3.5 g/g was observed in 10 out of 16 patients. Serum albumin levels decreased from 40.0 g/L [38.0 – 42.0] to 29.5 g/L [26.0 – 35.0] (p-value < 0.001), and 5/16 patients had authentic nephrotic syndromes. Glomerular filtration rate was not significantly altered: 79 mL/min/1.73m^2 [55 – 115] vs 84 mL/min/1.73m2 [53 – 111] before relapse. Individual trajectories are represented in figure 1. When available (n=8), kidney biopsies at clinical relapse consistently demonstrated typical MN IgG and PLA2R-positive deposits (Supplementary Figure S2). Electron microscopy analysis could only be performed for one of these patients, revealing the concomitant presence of both ancient and recent deposits. In comparison to the initial biopsy, patients at relapse exhibited a higher frequency of stage 3 MN (6/8 (75%), p-value = 0.018), consistently demonstrated at least one globally sclerotic glomerulus (p-value = 0.007). A trend towards more frequent FSGS lesions (50% vs 23%, p-value = 0.3) was observed (Supp. Table 1). On top of IIF, ELISA testing for anti-PLA2R antibodies was performed for 7 patients. All patients tested below the 14RU/mL threshold (<2RU/mL in 3, equal to 2RU/mL in one, 4RU/mL in one and 10RU/mL in two patients). In three patients, anti-PLA2R serology eventually became

				positive with IIF ratios between 1/50 and 1/500, ELISA between 22 and 75, 2 to 6 months after clinical relapse.
		(b) Indicate number of participants with missing data for each variable of interest	Table 1	
		(c) *Cohort study*—Summarise follow-up time (eg, average and total amount)	4	was reported during the 34 months [15 – 58] median follow-up.
Outcome data	15*	*Cohort study*—Report numbers of outcome events or summary measures over time	*4*	Twelve months after treatment, initiation median UPCR decreased from 3.70g/g [2.41 – 5.16] to 0.69g/g [0.52 – 0.95] (p value < 0.001) and median serum albumin increased from 29.5 g/L [26.0 – 35.0] to 38.0g/L [35.8 – 40.0] (p value < 0.001) (figure 1 A-B). eGFR remained stable: 83mL/min/1.73m^2 [54 – 107] vs 88mL/min/1.73m^2 [66 – 117] (p value = 0.8).
		Case-control study—Report numbers in each exposure category, or summary measures of exposure	*N/A*	
		Cross-sectional study—Report numbers of outcome events or summary measures	*N/A*	
Main results	16	(*a*) Give unadjusted estimates and, if applicable, confounder-adjusted estimates and their precision (eg, 95% confidence interval). Make clear which confounders were adjusted for and why they were included	3-4	All patients reached partial remission, and 3 patients (all treated with rituximab) achieved complete remission. No severe adverse event (defined by any event – infectious of not – that would lead to hospitalization, severe alteration of general condition, acute kidney failure or death) was reported during the 34 months [15 – 58] median follow-up. Seven out of 16 patients eventually reached complete remission (6 in the rituximab group, and 1 in the supportive care group). Five patients suffered another

			relapse after treatment within a median delay of 39 months [30 – 44].
	(*b*) Report category boundaries when continuous variables were categorized	N/A	
	(*c*) If relevant, consider translating estimates of relative risk into absolute risk for a meaningful time period	N/A	

Continued on next page

Other analyses	17	Report other analyses done—eg analyses of subgroups and interactions, and sensitivity analyses	N/A	
**Discussion**				
Key results	18	Summarise key results with reference to study objectives	4	We report a peculiar group of patients who, after successful treatment of PLA2R-associated membranous nephropathy, presented clinical relapse without detection of circulating anti-PLA2R antibodies by IIF at clinical relapse.<br><br>When considered individually, the proteinuria results could suggest FSGS scarring. However, the concomitant hypoalbuminemia, and moreover, the integration of these findings within the individual patients' timelines, along with the pronounced impact of rituximab on the depicted curves in Figure 1, strongly supports the presence of active immunological glomerular injuries. This is corroborated by the systematic positivity of the PLA2R glomerular staining whenever a biopsy was performed.
Limitations	19	Discuss limitations of the study, taking into account sources of potential bias or imprecision. Discuss both direction and magnitude of any potential bias	4	Failure to detect circulating anti-PLA2R could be explained by ELISA and IIF sensitivity limitations. While Western blot is considered more sensitive, it is not feasible for routine clinical use and was unavailable in this retrospective study.6 IIF may be more sensitive than ELISA but is prone to interpretative bias, especially at low titers. [...]Given the high prevalence of relapse in MN patients and the low likelihood of developing concurrent nephrotic disease, this reduction in specificity

				is unlikely to compromise the positive predictive value, supporting consideration of a lower ELISA threshold for relapse diagnosis. Unfortunately, ELISA is not routinely performed in IIF-negative cases here, limiting our ability to determine if more than 4 of the 7 would have tested positive at a lower threshold.
Interpretation	20	Give a cautious overall interpretation of results considering objectives, limitations, multiplicity of analyses, results from similar studies, and other relevant evidence	4	The "glomerulus as a sink" hypothesis, which proposes that glomerular antigenic sites must be saturated before circulating antibodies can be detected, has been proposed to explain the discrepancies between serological and histological testing at the first flare.9 There is no reason why this hypothesis should not also apply to relapsing patients.
Generalisability	21	Discuss the generalisability (external validity) of the study results	4	Large cohorts-based risk stratification scores have allowed international consensus on initial treatment strategies.1 Efforts should now be focused on relapsing patients to enhance the prediction of spontaneous remission probability and long-term risks, enabling personalized evidence-based treatment strategies.
**Other information**				
Funding	22	Give the source of funding and the role of the funders for the present study and, if applicable, for the original study on which the present article is based	N/A	

*Give information separately for cases and controls in case-control studies and, if applicable, for exposed and unexposed groups in cohort and cross-sectional studies.

Note: An Explanation and Elaboration article discusses each checklist item and gives methodological background and published examples of transparent reporting. The STROBE checklist is best used in conjunction with this article (freely available on the Web sites of PLoS Medicine at http://www.plosmedicine.org/, Annals of Internal Medicine at http://www.annals.org/, and Epidemiology at http://www.epidem.com/). Information on the STROBE Initiative is available at www.strobe-statement.org.
